# Comparative study of lumbar bone mineral content using DXA and CT Hounsfield unit values in chest CT

**DOI:** 10.1186/s12891-023-06159-6

**Published:** 2023-02-04

**Authors:** Dong-Ha Lee, MinWoo Kim

**Affiliations:** 1 Department of Orthopedic Surgery, Busan Medical Center, Busan, Republic of Korea; 2 School of Biomedical Convergence Engineering, Pusan National University, Yangsan, Republic of Korea

**Keywords:** Dual-energy x-ray absorptiometry (DXA), Computed tomography Hounsfield unit (CT HU), Bone mineral content (BMC), Morphometric texture analysis, Linear regression

## Abstract

**Background:**

Bone mineral content (BMC) values in certain bones and changes in BMC over time are key features for diagnosing osteoporosis. This study examined those features using morphometric texture analysis in chest computational tomography (CT) by comparing a dual-energy X-ray absorptiometry (DXA)-based BMC. An accessible approach for screening osteoporosis was suggested by accessing BMC using only Hounsfield units (HU).

**Methodology:**

The study included a total of 510 cases (255 patients) acquired between May 6, 2012, and June 30, 2020, at a single institution. Two cases were associated with two chest CT scans from one patient with a scan interval of over two years, and each scan was followed soon after by a DXA scan. Axial cuts of the first lumbar vertebra in CT and DXA-based L1 BMC values were corrected for each case. The maximum trabecular area was selected from the L1 spine body, and 45 texture features were extracted from the region using gray-level co-occurrence matrices. A regression model was employed to estimate the absolute BMC value in each case using 45 features. Also, an additional regression model was used to estimate the change in BMC between two scans for each patient using 90 features from the corresponding cases.

**Results:**

The correlation coefficient (CC) and mean absolute error (MAE) between estimates and DXA references were obtained for the evaluation of regressors. In the case of the BMC estimation, CC and MAE were 0.754 and 1.641 (g). In the case of the estimation of change in BMC, CC and MAE were 0.680 and 0.528 (g).

**Conclusion:**

The modality using morphometric texture analysis with CT HUs can indirectly help screening osteoporosis because it provides estimates of BMC and BMC change that show moderate positive correlations with DXA measures.

## Introduction

Osteoporosis, a common bone disease, is a bone metabolism disorder characterized by diminished bone strength and mass, resulting in an increased risk of fractures containing high components of trabecular bone such as the proximal femur, vertebral body of spine, and distal radius [1–3]. Therefore, it is important to establish a diagnostic system that can diagnose and prevent osteoporosis in advance. Currently, dual-energy X-ray absorptiometry (DXA) is the gold standard in measuring bone mineral density (BMD) [4, 5]. However, because BMD changes depending on the progression of osteosclerosis due to degenerative changes or vascular calcification and the degree of fat, careful interpretation of DXA is required [6–8]. This tendency is higher in the lumbar spine, which is examined through the abdomen; therefore, it is often more difficult to accurately measure BMD than femoral neck bone density [8].

Bone mineral content (BMC) is widely used as the most important material for bone fragility, strength, and structure; therefore, it plays an important role in predicting fractures. Since the trabecular bone has a high bone turnover rate, it is a bone structure that reflects bone evaluation the most by responding sensitively to metabolic stimuli. BMC covers trabecular bone that carries high potential accuracy of indicators of osteoporosis and fracture risk. In addition, Matkovic et al. found that true bone density does not provide accurate bone status concerning growth because the bone is a metabolically active organ [9]. Especially, in a growth and aging experiment, it is important to consider BMC, as opposed to bone density [10].

Measuring BMC by true bone density using empirical methods is not appropriate way since it cannot show all the remodeling phases of the bone [11, 12]. Therefore, additional tests that use bone marker assays and various radiological techniques to measure bone mineral content have recently emerged [13]. With the development of radiologic analysis, studies on bone quality analysis have been conducted using morphometric texture analysis with images such as CT and QCT [14]. However, despite its clinical value, CT screening is still not successfully commercialized because it is not sufficiently accurate for disease identification. Most previous studies focused on predicting BMD using DXA and they had technical limitations in that they only accounted for the mean HU value as a statistical feature [15–17]. To our knowledge, few studies assessed BMC using CT in the lumbar bone mineral.

Osteoporosis screening using CT is potentially valuable because it can increase screening rates without additional radiation exposure or patient costs. In addition, it is possible to automatically select potential patients and facilitate their management by storing CT images, previously taken for other clinical purposes, in a database. If BMC information along with BMD can be predicted from CT, it can increase the scope of CT usage for monitoring bone.

Therefore, this study aimed to establish an objective basis for quantifying the degree of BMC at a scan time point and BMC change over time using CT images. Specifically, we quantitatively analyze the HU values in CT, extract model-based texture features using gray level co-occurrence matrix (GLCM) and finally developed linear regression models using a combination of features with the guidance of DXA measures. Also, the least absolute shrinkage and selection operator (LASSO) was adopted for identifying the contribution of every feature to the estimation.

## Materials and methods

### Subjects for the region of interest

**Fig. 1:**
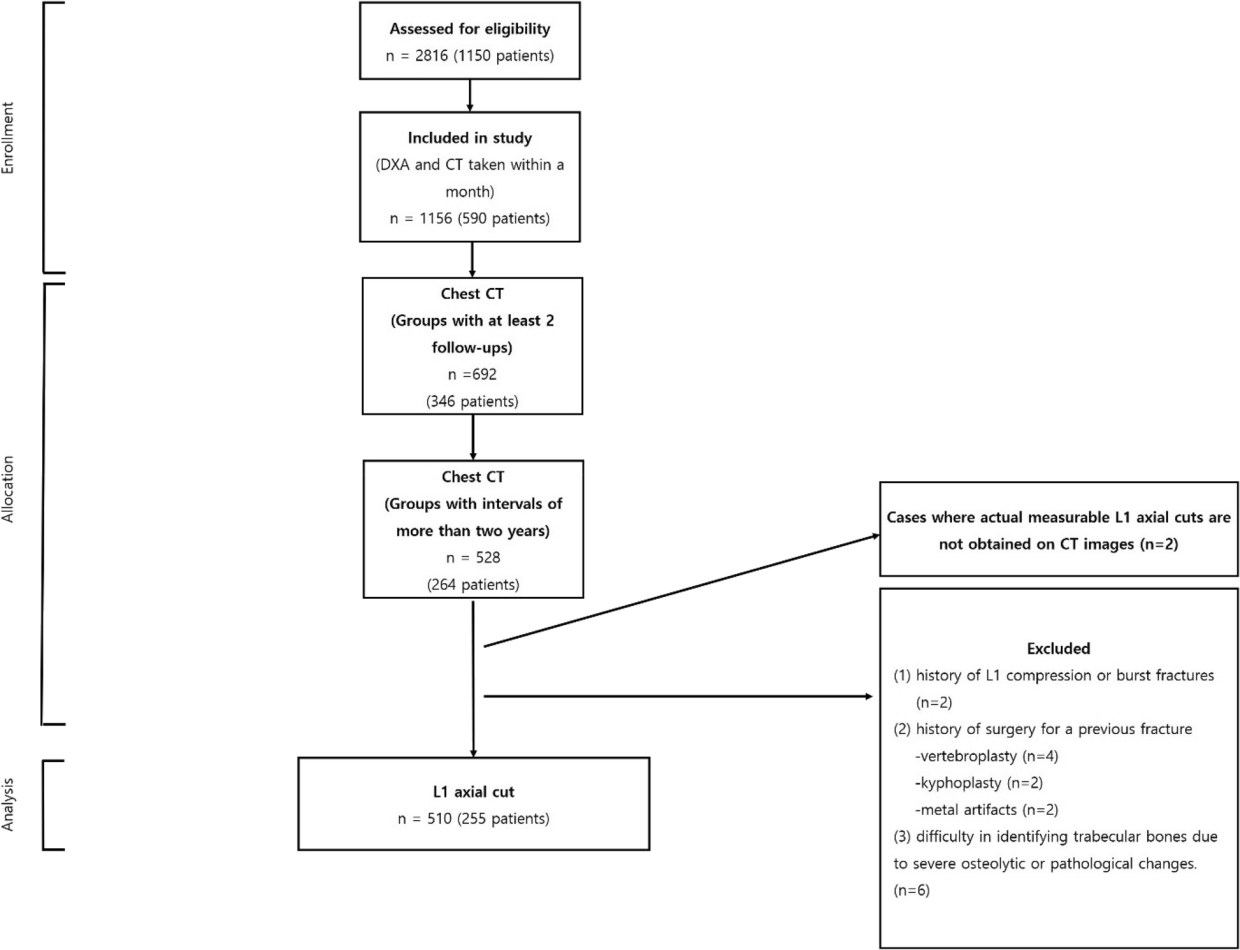
Flowchart showing the selection of L1 axial cut

The institutional review board (P01-202109-21-014) approved this study. Initially, a total of 2816 cases involving 1150 patients were collected. They had both CT and DXA in a single institution between May 6^th,^ 2012 and June 30^th,^ 2021. Among them, we selected 528 cases involving 264 patients with [1] less than a month gap between CT and DXA scan dates, [2] two or more follow-ups, and [3] more than a two-year interval between the first scan and follow-ups. Subsequently, we excluded 18 cases (9 patients) that met at least one of the following criteria: [1] CT image containing no actual measurable axial L1 cut (first lumbar vertebrae body axial cut), [2] history of L1 compression or burst fracture, [3] history of surgery for a previous fracture (e.g., vertebroplasty or kyphoplasty due to an L1 compression fracture, metal artifacts due to unstable burst fractures, etc.), and [4] difficulty in identifying trabecular bones due to severe osteolytic or pathological changes. Finally, 510 cases (255 patients) were included for analysis (Fig. 1).

### Imaging protocols of CT and DXA

A Siemens scanner (SOMATOM 128, Definition AS+; Siemens Healthcare, Forchheim, Germany) was used for the CT scans. For every scan, the protocol was a single-energy CT with 120 kVp, 247 mA, and a dose modulation of 0.6 mm collimation. The effective pitch was 0.8 and the reconstruction kernel was B60 (sharp). Reconstructed slice thicknesses were set at 5.0 mm for chest CTs (non-contrast). For the DXA scans, a standard device with a standard protocol (GE Lunar Prodigy, GE Healthcare) was used, and reports were obtained using vendor-specific software (Physicians Report Writer DX; Hologic, Discovery WI, USA).

**Fig. 2:**
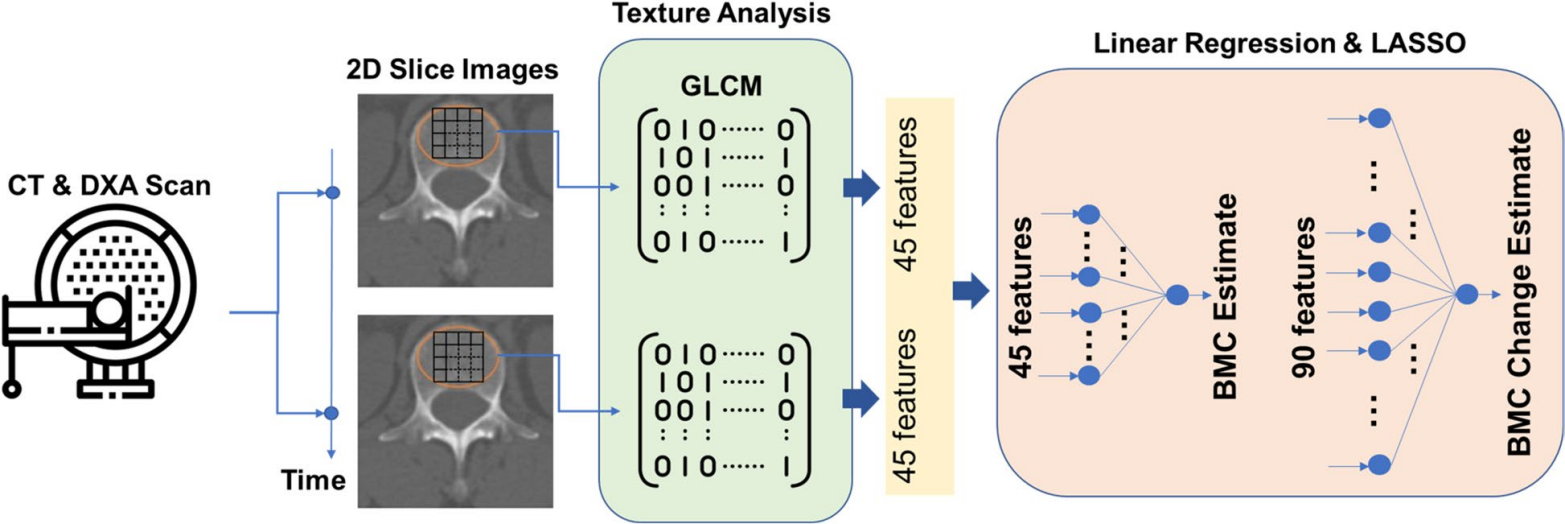
Schematic flow for BMC estimations from computed tomography. BMC, bone mineral content

### Estimation of BMC change using CT

Figure 2 illustrates the schematic flow of BMC estimations. Specifically, from the CT axial cuts of every patient, we selected one slice image that contained the maximum axial trabecular area of the bone. A total of 45 feature values ${\left\{{x}_{j}\right\}}_{j=1,.,45}$ were obtained from each area, where the five features were based on an intensity histogram (of CT HU values), and the others were based on a gray-level co-occurrence matrix (GLCM), which is widely used in texture analysis. The GLCM functions (Table 1) characterize the texture of an image by extracting statistical measures from a matrix that represents how often pixel pairs with specific values occur in an image [18, 19]. As shown in Table 1, we used multiple statistics ($k$) in the histogram and multiple combinations of directions ($l$), levels ($m$) and statistics ($n$) in GLCM. The feature index was given as $j=k+n+5\left(m-1\right)+20(l-1)$. Since each patient had two axial cuts of the chest CT, it provided a total of 90 features. We used a MATLAB function for generating a nonsymmetric version of matrices.

Two linear regressors were developed for extracting BMC information from CT. The first regressor was fed with 45 features from 1 case for estimating a BMC value at each scan time point. Meanwhile, the second regressor was fed with 90 features from 2 sequential cases from each patient for specializing in a change in BMC. Each regression provides an estimate as a linear sum of the features and one bias as ${\widehat{y}}_{j}={\sum }_{j=1}^{J}{w}_{j}{x}_{j}+b$, where ${x}_{j}$ denotes the $j$th feature. The optimal parameters are obtained as $\left\{{w}_{j}^{*},{b}^{*}\right\}=\underset{\{{w}_{j},b\}}{\text{argmin}}{\sum }_{i=1}^{I}{\left({y}_{i}-{\widehat{y}}_{i}\right)}^{2}$, where ${y}_{i}$ denotes the DXA reference.

In addition, LASSO was adopted for enhancing interpretability of the regression models. LASSO uses the ${l}_{1}$ penalty for sparsity as $\left\{{w}_{j}^{*},{b}^{*}\right\}=\underset{\{{w}_{j},b\}}{\text{argmin}}{\sum }_{i=1}^{I}{\left({y}_{i}-{\widehat{y}}_{i}\right)}^{2}+\lambda {\sum }_{i=1}^{I}\left|{x}_{i}\right|$, where $\lambda$ denotes the degree of the ${l}_{1}$ regularization. The larger $\lambda$, the sparser the final solution $\left\{{w}_{j}^{*}\right\}$.

**Table 1:** Gray-level co-occurrence matrix feature parameters

Analytical tool	Parameter	Value/name/function	Feature #
Histogram	Statistics (k)	mean (k = 1), standard deviation (k = 2), skewness (k = 3), kurtosis (k = 4) entropy (k = 5)	5
Texture (GLCM)	Directions (l)	horizontal (l = 1), vertical (l = 2)	2 × 4 × 5 = 40
	Levels (m)	16 (m = 1), 32 (m = 2), 64 (m = 3), 128 (m = 4)	
	Statistics (n)	contrast (n = 1), correlation (n = 2), energy (n = 3), homogeneity (n = 4), variance (n = 5)	

## Results

### Patient demographics

In total, 255 patients (122 men and 133 women) were included for analysis. The mean age of the patients was 52.51 ± 8.56 years, and the average BMI index was 23.79 ± 4.65 kg/m^2^. The time between chest CT and DXA was 0.89 ± 5.22 days. The average interval between the first and last chest CTs was 1066 ± 24.57 days. In addition, the average interval between the first and last DXAs was 1048 ± 21.68 days. The patient demographics are summarized in Table 2.

**Fig. 3:**
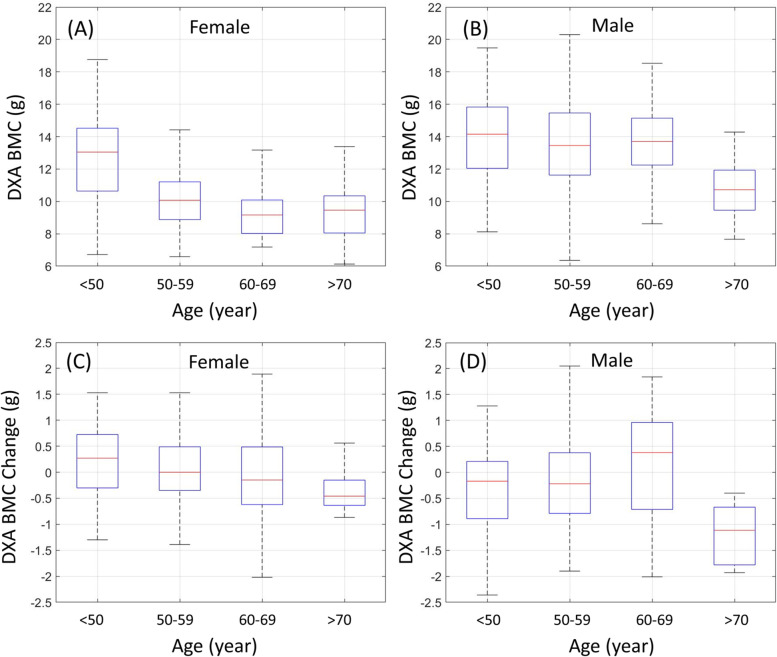
Statistics of DXA measures using box plots. The central red line on each box indicates the median, and the bottom and top edges of the box indicate the 25th and 75th percentiles, respectively. **A** and **B** show the statistics of BMC (at one scan time point) over the age range in the female and male groups, respectively. **C** and **D** show the statistics of BMC change (between two scan time points) over the age range in female and male groups, respectively

The statistics of DXA measures are displayed in Fig. 3. Figure 3 (A) shows the BMC over age range in the female group. The median values at the ranges, < 50, 50–59, 60–69, and > 70, were 13.04, 10.07, 9.16, and 9.46, respectively. The differences between BMCs at the range < 50 and other ranges were statistically significant (p-value < 0.05). Figure 3 (B) shows the BMC over the age range in the male group. The median values at the ranges, < 50, 50–59, 60–69, and > 70, were 14.15, 13.45, 13.70, and 10.72, respectively. Overall, the values in the male group were higher than those in the female group. In the male group, the differences between BMCs at the range > 70 and other ranges were statistically significant (*p*-value < 0.05). Figure 3 (C) and (D) show the BMC change (between two scan time points) over the age range in the female and male groups, respectively. In the female group, the median values at the ranges, < 50, 50–59, 60–69, and > 70, were 0.27, 0.00, -0.15, and − 0.46, respectively. In the male group, the medians at the ranges were − 0.17, -0.22, 0.38, and − 1.11, respectively, and the differences between BMC changes at the range > 70 and other ranges were statistically significant (*p*-value < 0.05).

**Fig. 4:**
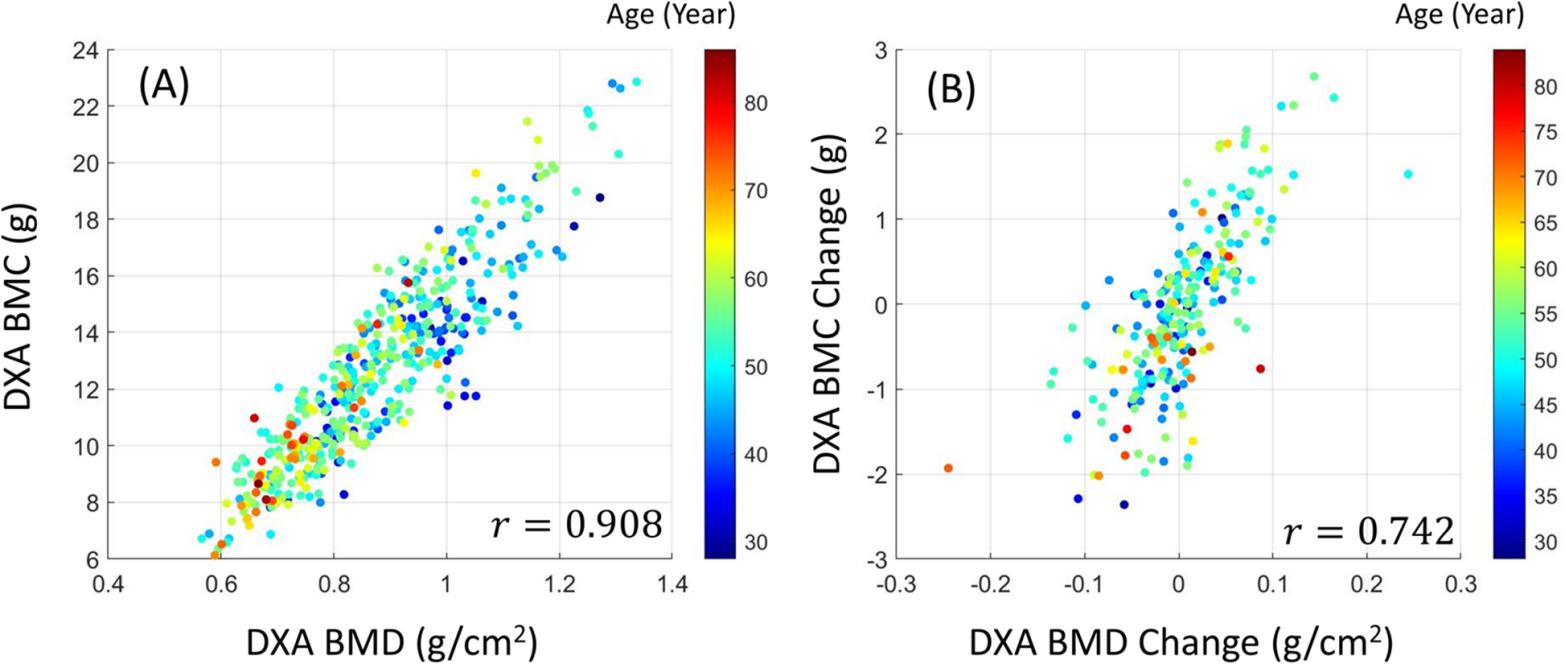
Correlation between DXA BMC and DXA BMD measures using scatter plot. The color of each marker represents the age of a patient sample. The metric $r$ denotes the Pearson correlation coefficient. **A** plots BMC measures from 510 cases. **B** plots BMC changes from 255 patients

**Fig. 5:**
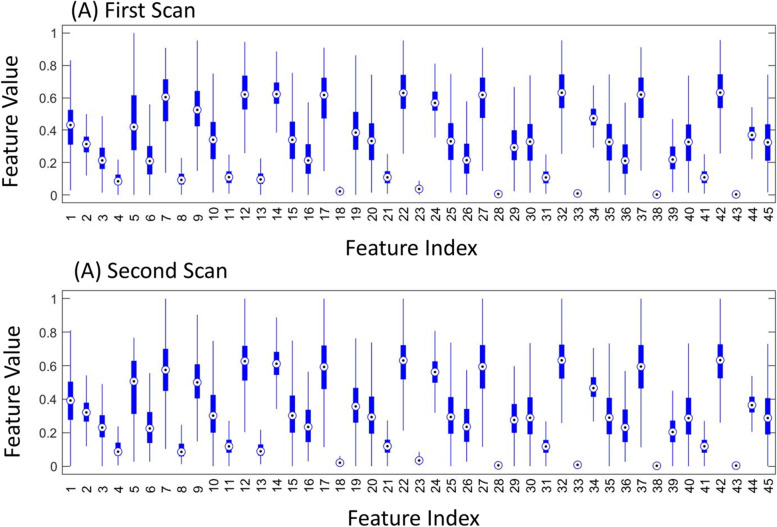
Sample statistics for each feature using box plots. The central red line on each box indicates the median, and the bottom and top edges of the box indicate the 25th and 75th percentiles, respectively. Every feature was min-max normalized. **A** and (**B**) show the sample statistics for the first and second scans, respectively

Figure 4 shows the relationship between DXA BMC and DXA BMD measures using a scatter plot. BMC absolute and BMC change were considerably correlated with BMD absolute and BMD change, respectively. The Pearson correlation value $r$ between BMC and BMD was 0.908, and the correlation value between BMC change and BMD change was 0.742. Figure 5 shows the statistics of (min-max) normalized feature samples in the texture analysis.

**Table 2:** Demographic data of study participants

Case (number)	510 (255)
Mean age (years)	52.51 ± 8.56
The time between CT and DXA dates (days)	0.89 ± 5.22
The interval between the first CT and last CT (days)	1066 ± 24.57
The interval between the first DXA and last DXA (days)	1048 ± 21.68
Sex (male/female)	122/133
BMI (kg/m^2^)	23.79 ± 4.65

### Correlation test

**Fig. 6:**
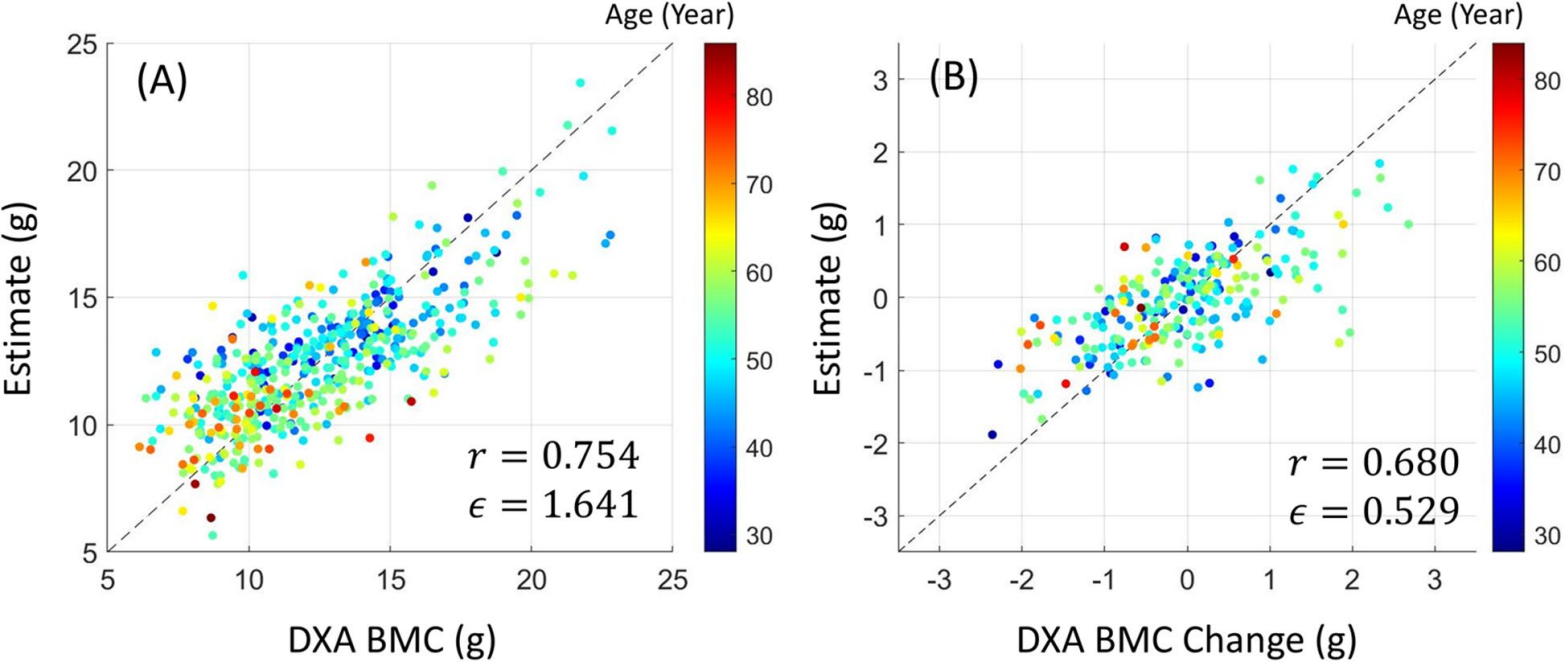
Linear regressor estimation results. The color of each marker represents the age of a patient sample. **A** and (B) show the estimates of BMC and BMC change, respectively, over DXA references. The metrics $r$ and $\mathit\in$ denote the correlation coefficient and mean absolute error, respectively

As described in Sect. 2.3, feature extractions and linear regressions were applied to obtain estimates from only CT images. Figure 6, (A) and (B) show scatter plots representing estimates of BMC absolute and BMC change, respectively. In the regression for BMC absolute, the correlation coefficient and mean absolute error between the estimates and references were 0.754 and 1.641 (g), respectively. In the regression for BMC change, the correlation coefficient and mean absolute error between the estimates and references were 0.680 and 0.528 (g), respectively. The p-values in paired t-tests were 1 because the linear regressor is an unbiased estimator.

**Fig. 7:**
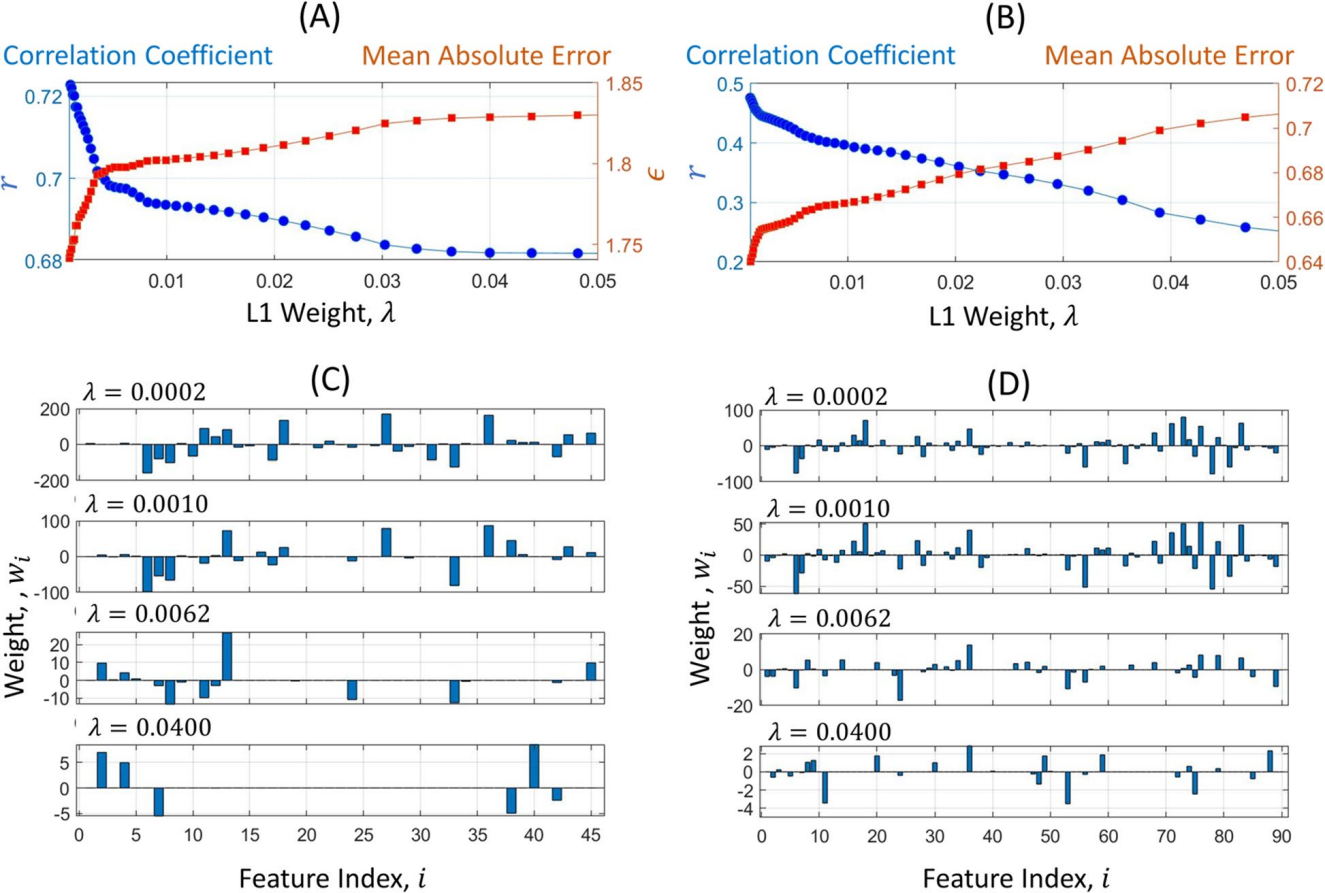
LASSO results. **A** and (**B**) show the correlation coefficient and mean absolute error between estimate and DXA reference over the degree of the ${\varvec{l}}_{1}$ regularization, $\varvec{\lambda }$. **C** and (**D**) show the weights in the LASSO regressor when $\varvec{\lambda }$ is 0.0002, 0.0010, 0.0062 and 0.0400. **A** and (**C**) are the results of BMC regressor, and (**B**) and (**D**) are the results of BMC change regressor

Figure 7 shows the results of LASSO regressors for BMC absolute ((A) and (C)) and BMC change ((B) and (D)). The correlation coefficient decreased, and the mean absolute error increased as the degree of the ${l}_{1}$ penalty increased as shown in Fig. 7 (A) and (B). Due to the penalty, many weights were constrained to be zeros as shown in Fig. 7 (C) and (D). In the BMC regressor, the degree of the penalty led to the gentle performance loss for the sparsity of weights. When the degree ($\lambda$) was 0.04, the regressor provided around 0.68 as a correlation coefficient using only 6 features. On the other hand, in the BMC change regressor, the penalty caused a relatively high-performance loss. It seems that each feature was independently involved in this estimation, and it was hardly substituted with other features.

## Discussion

This study conducted texture analysis using CT HUs to create linear regression models using texture features to estimate BMC and BMC change, and the correlation between these estimates and actual DXA values was assessed. For L1, the regressors provided 0.754 and 0.680 for BMC and BMC change, respectively, as correlation coefficients. The main strength of this study is that the results are robust. Compared to previous studies, we used a larger sample size, which included both male and female patients, and the scan interval (0.89 ± 5.22 days) between chest CT and DXA was shorter [20]. In addition, through texture analysis of CT HU values, we derived models that estimated the degree of BMC value or changes in BMC values that were highly correlated with those of DXA-based BMC. We believe that these results provide an academic basis for using only CT to estimate BMC in bones other than the spine and femur.

The BMC change can imply the possibility of osteoporosis or medications that cause secondary osteoporosis such as hyperparathyroidism, vitamin D deficiency, celiac disease, steroids, and aromatase inhibitors [21, 22]. Thus, by tracking the BMC change from past to current, it can suggest proper education and treatment to patients. Moreover, the estimation using CT is beneficial especially in the group that rarely takes DXA scans because of insurance limitations (ex. non-menopause women under age 65 and men under age 70 in Korea). If CT is taken for regular health checkups or other screening purposes, the latent osteoporosis can be screened as opportunistic CT imaging.

In this study, chest CT (non-contrast) was selected as the base for texture analysis because it is often performed for health examination purposes and because DXA for osteoporosis diagnosis is also performed around the same time. In particular, chest CT was selected in this study because it also includes the ROI (L1), which scans the DXA for BMD measurement.

Smith et al. [23] reported that the variance of bone mineral values does not increase with aging and that individuals exhibit significantly different rates of loss over time [24]. Although bone mineral value and aging do not show a negative linear correlation, bone mineral content does decrease with age [24]. In this study, it was found that the bone mineral content decreases over time, as shown in Fig. 3, where negative and positive signs indicate a decrease and increase, respectively.

The usefulness of CT texture analysis over the DXA method for measuring bone mineral content is that this method can distinguish the trabecular from the cortical part of the bone. Since the weak regions of the vertebrae are the upper and anterior parts, where low density can be misinterpreted by other higher-density structures (25), CT texture analysis can overcome the limitations of empirical methods. Therefore, the risk assessment for fractures can potentially be optimized using CT texture analysis to measure not only the whole body of the vertebrae but also its weakest point, the upper and foremost parts of the vertebrae.

However, these findings should be interpreted with caution because of the following limitations. First, information on patients’ osteoporosis risk factors was excluded in the process of collecting demographic data. Major modifiable risk factors, such as inadequate nutritional absorption, smoking, and binge alcohol drinking, were not investigated during the follow-up period. In addition, no investigations were conducted on the presence or absence of secondary causes of osteoporosis. Second, we did not check the use of steroids, immunosuppressants, antiepileptic drugs, heparin, gonadotropic agents or antagonists, cancer chemotherapy drugs, etc. which may accelerate changes in BMC [25, 26]. Third, since it was used as the ROI model of the chest CT, only the ROI for the L1 axial cut could be obtained; therefore, it was not possible to compare the quantitative measurements of the trabecular bone of other lumbar spine bodies with the amount of each BMC. Fourth, as the follow-up periods were short (CT interval: 1066 ± 24.57, DXA interval: 1048 ± 21.68), studies on BMC changes have not been conducted over a long period. Fourth, our results using CT HU texture analysis were not compared with those of QCT. QCT can overcome the drawbacks of DXA through quantitative approaches to bone quality assessment. However, it is not routinely adopted because of its limited applicability to the spine, phantom calibration requirement, high cost, and high radiation risk [27–29]. Nevertheless, further comparative studies using QCT are needed to confirm our findings. Lastly, because the CT and DXA images were acquired predominantly at a single institution, data bias could be a problem.

Finally, we established the ROI of the desired location after the CT scan, creating a model to predict BMC change as well as BMC using CT HU texture analysis. This also means that this model can be further studied by establishing a framework for inferring BMC values using CT, rather than general DXA image locations such as the lumbar spine or hip.

## Conclusion

The modality using morphometric texture analysis with CT HUs can indirectly help in screening osteoporosis because it provides estimates of BMC and BMC change that show moderate positive correlations with DXA measures.

## Data Availability

The datasets are available from the corresponding author on reasonable request.
